# Remote Digital Measurement of Facial and Vocal Markers of Major Depressive Disorder Severity and Treatment Response: A Pilot Study

**DOI:** 10.3389/fdgth.2021.610006

**Published:** 2021-03-31

**Authors:** Anzar Abbas, Colin Sauder, Vijay Yadav, Vidya Koesmahargyo, Allison Aghjayan, Serena Marecki, Miriam Evans, Isaac R. Galatzer-Levy

**Affiliations:** ^1^AiCure, New York, NY, United States; ^2^Adams Clinical, Watertown, MA, United States; ^3^Karuna Therapeutics, Boston, MA, United States; ^4^Psychiatry, New York University School of Medicine, New York, NY, United States

**Keywords:** major depressive disorder, Montgomery-Åsberg Depression Rating Scale, machine learning, computer vision, digital biomarker, antidepressant treatment, digital phenotyping

## Abstract

**Objectives:** Multiple machine learning-based visual and auditory digital markers have demonstrated associations between major depressive disorder (MDD) status and severity. The current study examines if such measurements can quantify response to antidepressant treatment (ADT) with selective serotonin reuptake inhibitors (SSRIs) and serotonin–norepinephrine uptake inhibitors (SNRIs).

**Methods:** Visual and auditory markers were acquired through an automated smartphone task that measures facial, vocal, and head movement characteristics across 4 weeks of treatment (with time points at baseline, 2 weeks, and 4 weeks) on ADT (*n* = 18). MDD diagnosis was confirmed using the Mini-International Neuropsychiatric Interview (MINI), and the Montgomery–Åsberg Depression Rating Scale (MADRS) was collected concordantly to assess changes in MDD severity.

**Results:** Patient responses to ADT demonstrated clinically and statistically significant changes in the MADRS [*F*_(2, 34)_ = 51.62, *p* < 0.0001]. Additionally, patients demonstrated significant increases in multiple digital markers including facial expressivity, head movement, and amount of speech. Finally, patients demonstrated significantly decreased frequency of fear and anger facial expressions.

**Conclusion:** Digital markers associated with MDD demonstrate validity as measures of treatment response.

## Introduction

Patients with major depressive disorder (MDD) are heterogeneous in both their clinical presentation and their response to antidepressant treatment (ADT) (1, 2). It is theorized that treatment effects may be obfuscated because MDD measurements combine heterogeneous symptoms that reflect distinct neurobiological and social processes while pharmacological treatments target specific neurobiological processes such as serotonergic tone. For example, patients with different subtypes of MDD, such as cognitive and neurovegetative phenotypes, have demonstrated differential treatment response to distinct classes of ADTs (3, 4). As such, there are significant efforts to refocus treatment research on measures that match the underlying neurobiological treatment target (5). Disentangling the heterogeneity in MDD can lead to better risk and treatment response assessment by shifting the focus of investigation to narrow phenotypes that reflect the underlying neurological deficit and target of treatment (5, 6).

The use of digital measurements that relate to underlying biological phenotypes, termed digital phenotyping (7), has been proposed as a methodology to improve measurement of underlying illness by capturing digital proxy measures of clinical functioning. An example of digital phenotyping is the measurement of activity as a proxy measure of mood or anxiety states using actigraphy or geolocation captured from an individual's smartphone (8, 9). While novel measurements are promising, validation is required before such metrics can be interpreted clinically. The key steps to validation include comparison with traditional clinical measures, both cross-sectionally and as they change with the disease or treatment course (10). Such measures should strive for ease of collection and increased sensitivity to facilitate frequent, accurate assessment and should be validated in relation to narrower biological phenotypes and treatment targets than those that traditional endpoints assess. This will ultimately lead to improved, dynamic treatment research and clinical decision making (9) based on modulation of underlying neurobiological deficits (11).

Based on prior knowledge, visual, and auditory data sources represent a compelling direction for objective measurement of patient functioning in MDD. Beginning with observations by Emil Kraepelin, patients with depression have been shown to produce slowed and spaced out speech, where they appear to “become mute in the middle of a sentence” and demonstrate altered facial behavior, regarding which he states, “the facial expression and the general attitude are sleepy and languid” (12). These clinical observations by Kraepelin have been corroborated and extended with standardized methods to assess facial expressions, vocal characteristics, and movement patterns using audio and video data sources. The same paucity of speech has been observed in acutely suicidal patients (13). Indeed, both speech and facial/bodily movement represent sensitive biological outputs that change with physiological and cognitive variability (13–15).

A number of visual and auditory characteristics that correspond to known MDD symptoms can now be directly quantified. This includes reduced gross motor activity (16), slumped posture (17), reduced head movement variability (17–19), reduced facial expressivity (20), reduced speech production (21), and increased negative affect (22, 23). The automated measurement of these clinical features introduces the possibility of objective automated assessment. Given that audio and video data sources can be captured remotely, this further introduces the possibility of greatly scaling the reach and frequency of assessment. Increased scale and objectivity can facilitate increased accuracy and accessibility of clinical risk and treatment response assessments.

Serotonin signaling deficits represent a primary biological target for treatment in MDD. Serotonergic tone mechanistically impacts motor functioning directly through interactions with dopamine and norepinephrine signaling (24–26). Postmortem comparison of suicides compared with controls demonstrates significant reductions of brain serotonin (27, 28). More specific mapping of mRNA expression patterns demonstrates reduced expression of serotonin mRNA subtypes that are relatively widespread and other subtypes that are specific to the frontopolar cortex amygdala circuitry (29). This circuitry governs the expression and regulation of threat and anxiety (30).

In this exploratory pilot study, we tested the ability of digitally measured facial, vocal, and movement behaviors to measure depression severity and treatment response across 4 weeks of ADT in individuals with MDD. We hypothesized that overall facial expressivity, amount spoken, and head movement measured from video and audio captured during smartphone-based tasks would increase in response to ADT. We also hypothesized that negative facial affect (i.e., fear and anger) would decrease in response to treatment. In doing so, we aimed to evaluate the ability of remote, automated, digital assessments to measure depressive symptomatology with reliability and accuracy. We also hoped that findings from this pilot study would inform future studies with larger sample sizes that can delve further into how such measurements are affected in different MDD subpopulations and varying treatment regimens.

## Methods

### Study Participants

Participants were identified through advertisements posted on social media. Individuals who self-identified as experiencing depression were screened over the telephone to assess depression symptoms. Potentially eligible subjects were then scheduled for an in-person pre-screening visit with a clinician to assess primary eligibility criteria. Individuals who met the criteria and provided informed consent participated in a screening assessment with a psychological rater, which included the Mini-International Neuropsychiatric Interview (MINI), Structured Interview Guide for the Montgomery–Åsberg Depression Rating Scale (SIGMA-MADRS), Columbia Suicide Severity Rating Scale (C-SSRS), and the Quick Inventory of Depressive Symptomatology Self-Report (QIDS-SR16). All study activities were approved by an institutional review board.

To be included in the study, subjects had to meet *Diagnostic and Statistical Manual of Mental Disorders*, 5th Edition (DSM-5) criteria for single or recurrent MDD based on the MINI with a current major depressive episode of ≥8 weeks and a MADRS total score of ≥20. Participants must have also been, in the opinion of the study psychiatrist, medically stable and a good candidate for treatment with a monoamine ADT. Key exclusion criteria included significant medical complications (e.g., uncontrolled cardiac or endocrine disorders, and diagnosis or treatment for cancer within the past 2 years), significant psychiatric complications (e.g., other primary psychiatric diagnoses and substance use disorders), intellectual disability (though no participants had to be excluded based on this criteria), or the use of certain prohibited concomitant medications (e.g., prescription painkillers/opioids; though use of benzodiazepines was not an exclusion criterion, none of the study participants reported in this manuscript were on benzodiazepines). Subjects who endorsed active suicidal ideation with intent or recent suicidal behavior (within the past 6 months), or who, in the opinion of the investigator, were at significant risk for suicidal behavior were excluded.

Participants who met screening eligibility criteria subsequently completed a visit with a study psychiatrist and were prescribed an ADT consistent with standard of care. Participants who demonstrated significant decreases in depression severity, indicated by a 30% reduction in MADRS total score over 4 weeks of ADT, were included in the sample (*n* = 18). The sample included seven men and 11 women (mean age = 30.2 ± 8.6). The mean body mass index (BMI) was 28.7 ± 5.6. Baseline total MADRS scores ranged from 25 to 45 (mean = 34.1 ± 4.9). Five study participants (28%) were on ADT at the time of screening, and most (89%) had recurrent MDD. The mean major depressive episode duration was 11 months, ranging from 2 to 43 months.

### Treatment and Assessment Conditions

All patients were prescribed either a selective serotonin reuptake inhibitor (SSRI) or serotonin–norepinephrine uptake inhibitor (SNRI) at label-specified doses based on the clinician's discretion. Time elapsed between the first participant in and last participant out was 6 months. Treatment response was measured at biweekly intervals using two independent assessments described below.

### Assessments

#### Clinical Assessment

*Montgomery–Åsberg Depression Rating Scale*: The MADRS is a 10-item clinician administered scale for the measurement of MDD with validated clinical cut points for severe (>34), moderate (20–34), mild (7–19), and asymptomatic (<7) depression. The MADRS has demonstrated validity as a sensitive measure of ADT response (31). The MADRS was administered by trained psychological raters with prior scale experience at week 0 (baseline) and at ~2 and 4 weeks posttreatment initiation.

#### Remote Smartphone-Based Video Assessments

All participants were asked to download the AiCure app (AiCure, LLC, New York, NY www.aicure.com) on their personal smartphone for measurement of digital markers of MDD. They were then trained by the study team on how to use the app to participate in remote assessments. This software platform has historically been used in clinical research for reporting of patient behavior to clinicians, including medication adherence, electronic patient-reported outcomes, and ecological momentary assessments, with considerable work done on patient acceptance and usability (32, 33). An additional functionality of capturing video and audio in response to prompts (as described below) was utilized for the purposes of this study (34, 35).

Participants completed weekly remote assessments for the length of the study. The assessment consisted of a smartphone-based adaptation of a paradigm to examine emotional valence in response to varied emotional imagery (27, 28, 36). At each assessment time point, they were prompted to view images taken from the Open Affective Standardized Image Set (OASIS) (37). The image set has emotional valence scores for each image based on responses recorded from a large, heterogeneous population, with lower scores referring to negatively valenced images and higher scores referring to positively valenced images. The valence scores were z-scored, and images with resulting scores of −0.5 to 0.5 standard deviation from the mean were considered neutrally valenced, images with resulting scores <1.5 standard deviation from the mean were considered negatively valenced, and images with resulting scores >1.5 standard deviation from the mean were considered positively valenced. The space in standard deviations between the classifications was added to ensure adequate separation between the image valences while also ensuring that enough images were left in each class to allow for there to be no repetition of images presented to the patients over the course of the study.

**Figure 1:**
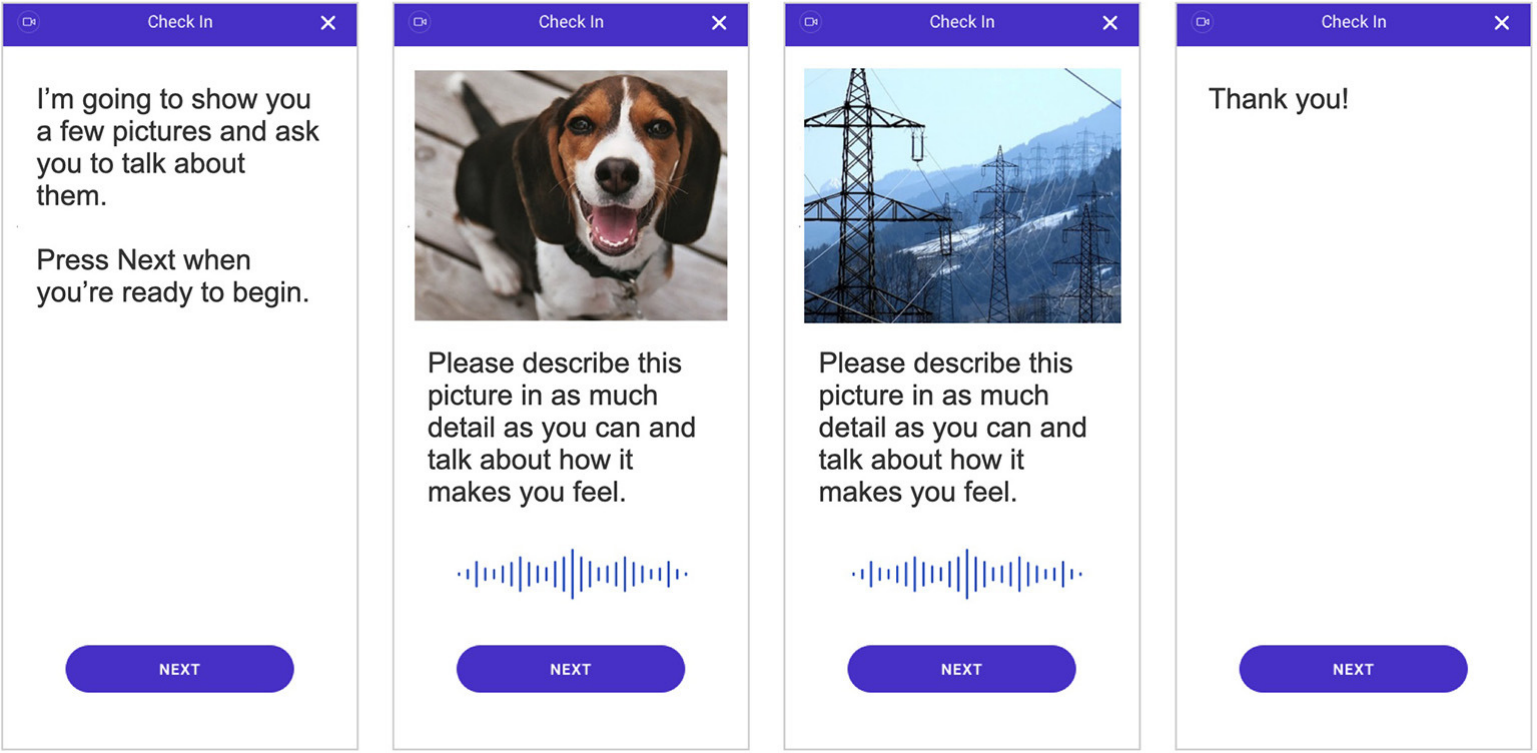
Depiction of the smartphone-based assessment that all individuals completed. Video and audio of participant responses were recorded during the assessment and used to quantify behavioral characteristics and subsequently measure digital markers of major depressive disorder (MDD) severity.

As part of the weekly remote assessments, patients were shown three positive images and three negative images padded with seven neutral images in between. The images were shown in series, starting with a neutral image, followed by a positive image, and then another neutral image before showing a negative image. This pattern was repeated until three positive and three negative images were shown and ended with a neutral image. This order was selected to avoid drastic shifts in image valences, i.e., switching directly between negative and positive images; by padding with neutral images, we hoped to alleviate any priming effects that may be present. For each image, the participant was asked to speak to the image by describing what they see in the picture and how it makes them feel (see Figure 1) and were required to speak for at least 10 s per image. Special care was also taken to ensure that participants were not shown the same image twice over the course of the study in order to limit any habituation effects of participating in the assessments.

### Digital Marker Calculation

Video and audio were captured continuously during the smartphone assessment using the smartphone front-facing camera and microphone. Data were uploaded and processed through Health Insurance Portability and Accountability Act (HIPAA)-compliant backend services for transfer and storage of protected health information (PHI). Video was extracted for analysis for the portion of the task where the participant is observing the image and responding to it. Both video and audio were extracted and analyzed for the portion of the task when the participant was describing the image.

All analyses were conducted in python with use of open-source tools. All digital biomarker variables analyzed were acquired through the use of OpenDBM, an open-source software package that combines tools for measurement of facial, vocal, and movement behaviors, developed partially for the research presented in this manuscript (https://github.com/AiCure/open_dbm). Code for all subsequent statistical analyses presented in this manuscript has also been made available online: https://github.com/AiCure/ms_dbm_adamsclinicalstudy. A total of 17 digital measurements in addition to the MADRS scores were used to measure response to treatment. A subset of the results from those comparisons is presented in the main text (Table 1). A full list of comparisons is provided Supplementary Table 1. There was no primary endpoint that was being analyzed as part of this study; rather, the ability of a set of digital markers (facial, vocal, and movement) was being analyzed individually, with the collective comparisons indicating the usefulness of digital measurement tools in general.

**Table 1:** Repeated-measures ANOVA results for all visual and voice markers measured in response to positive, negative, and neutral visual stimuli from baseline to 4 weeks of ADT.

	**Neutral stimuli**				**Positive stimuli**				**Negative stimuli**			
	**Sph**.	**F**	***p***	**+/–**	**Sph**.	**F/W**	***p***	**+/–**	**Sph**.	**F**	***p***	**+/–**
**Voice percentage**	**T**	**5.60**	**0.0095**	**+**	**T**	**3.59**	**0.0042**	**+**	**T**	**4.66**	**0.0187**	**+**
Anger intensity	T	13.28	<0.0001	+	T	21.19	<0.0001	+	T	19.96	<0.0001	+
Anger count	F	2.54	0.1214	n/a	F	0.92	0.3787	n/a	F	2.40	0.1307	n/a
Disgust intensity	T	12.00	0.0002	+	T	9.00	0.0009	+	T	9.43	0.0007	+
Disgust count	T	0.51	0.6033	n/a	T	1.33	0.2796	n/a	T	0.31	0.7358	n/a
Fear intensity	T	41.23	<0.0001	+	T	32.50	<0.0001	+	T	60.38	<0.0001	+
Fear count	F	4.84	0.0413	–	F	0.67	0.5182	n/a	F	0.77	0.4287	n/a
Happiness intensity	F	6.03	0.0232	+	F	5.21	0.0306	+	F	4.38	0.0445	+
Happiness count	T	0.03	0.9666	n/a	T	0.46	0.6362	n/a	F	1.72	0.2089	n/a
Sadness intensity	T	13.53	<0.0001	+	T	8.67	0.0012	+	T	10.54	0.0004	+
Sadness count	F	0.59	0.4690	n/a	T	2.05	0.1473	n/a	T	1.90	0.16815	n/a
Surprise intensity	T	22.29	<0.0001	+	T	17.31	<0.0001	+	T	26.10	<0.0001	+
Surprise count	T	0.14	0.8665	n/a	T	0.20	0.8194	n/a	T	0.16	0.8497	n/a
**Overall expressivity**	**T**	**32.60**	**<0.0001**	**+**	**T**	**40.67**	**<0.0001**	**+**	**T**	**36.95**	**<0.0001**	**+**
**Head movement mean**	**F**	**8.90**	**0.0069**	**+**	**F**	**3.58**	**0.0413**	**+**	**F**	**2.49**	**0.1335**	**n/a**
**Head movement standard deviation**	**F**	**3.68**	**0.0378**	**+**	**T**	**1.53**	**0.2333**	**n/a**	**F**	**1.59**	**0.2274**	**n/a**
**Head pose change mean**	**F**	**5.01**	**0.0325**	**+**	**T**	**3.18**	**0.0570**	**+**	**F**	**1.41**	**0.2595**	**n/a**

*In particular, overall facial expressivity, voice percentage, and head movement markers showed an overall increase in response to treatment, consistent with the decrease in MDD symptom severity. Sph. indicates sphericity assumption being true or false, F indicates F-statistic, p indicates p-value of F-statistic, and +/– indicates increased or decreased values compared with baseline (n/a indicates no significant change was observed)*.

*ADT, antidepressant treatment; MDD, major depressive disorder. Bold indicate the main findings*.

#### Facial Marker Calculation

First, all videos were segmented into individual video frames at 30 frames per second. Next, each frame was segmented into three matrices consisting of red, blue, and green spectrum pixels for use in computer vision (CV) modeling using OpenCV, an open-source CV software package (38). Subsequently, each frame was analyzed using OpenFace (39), an open-source software package that has demonstrated validity next to expert human ratings of Facial Action Coding System (FACS) (23), a standardized methodology to measure facial movements that reflect the activity in the underlying human facial musculature used in the production of basic emotions (i.e., *happiness, fear, anger, surprise, sadness*, and *disgust*).

Specifically, for each frame OpenFace outputs, (1) binary activation of each facial action unit (AU) was utilized to calculate the presence of facial emotions, and (2) the degree of expressivity for that AU was utilized to calculate intensity of facial emotions. From AU measurements, emotion behavior was calculated including (1) the presence or absence of each emotion for each frame selected as the most probable based on the observed AU activation, termed “count,” and (2) the level of activation for each emotion and across all emotions, termed “intensity.” Following the calculation of these variables for each frame, a set of variables was calculated that represented the count of emotions expressed across all frames divided by number of frames (*fear count, anger count, surprise count, sadness count, happy count*, and *disgust count*) and the intensity of emotion averaged over all frames (fear intensity, anger intensity, surprise intensity, sadness intensity, and disgust intensity). Additionally, a composite score of overall facial intensity summed across all emotions was calculated (*overall facial expressivity*).

#### Voice Marker Calculation

Recordings were segmented into speech and non-speech parts using parselmouth, an open-source software package that utilizes Praat software library (40) functions for vocal analysis (41). The ratio of speech to white space between words was calculated to represent the amount of time participants spoke compared with non-speech (*voice percentage*).

#### Movement Marker Calculation

For each frame of video, head position and angle were acquired using OpenFace. The average framewise displacement of the head between frames (*head movement mean*) and its standard deviation (*head movement standard deviation*) were calculated as measures of head movement. The mean change in angle of the head (*head pose change mean*) was calculated as an additional measure of head movement.

### Data Analysis

Change over time in MADRS and facial, voice, and movement variables (termed digital markers) was calculated using repeated-measures analysis of variance (ANOVA). To avoid capitalizing on change when doing multiple comparisons or testing for multiple hypotheses, *p*-values were corrected using false discovery rate (FDR) correction (42). The sphericity assumption, which is the condition where the variances of the differences between all combinations of related groups are equal, was formally tested for each ANOVA. When this assumption proved to hold, the F-statistic and corresponding *p*-value were used. When the sphericity assumption was violated, Mauchly's W statistic and corresponding *p*-value were used (43). Additionally, pairwise comparisons were calculated between each time point to determine where change across time points occurs (i.e., baseline to 2 weeks, baseline to 4 weeks, and 2–4 weeks) controlling for FDR using Tukey's test.

## Results

### Depression Response

**Figure 2:**
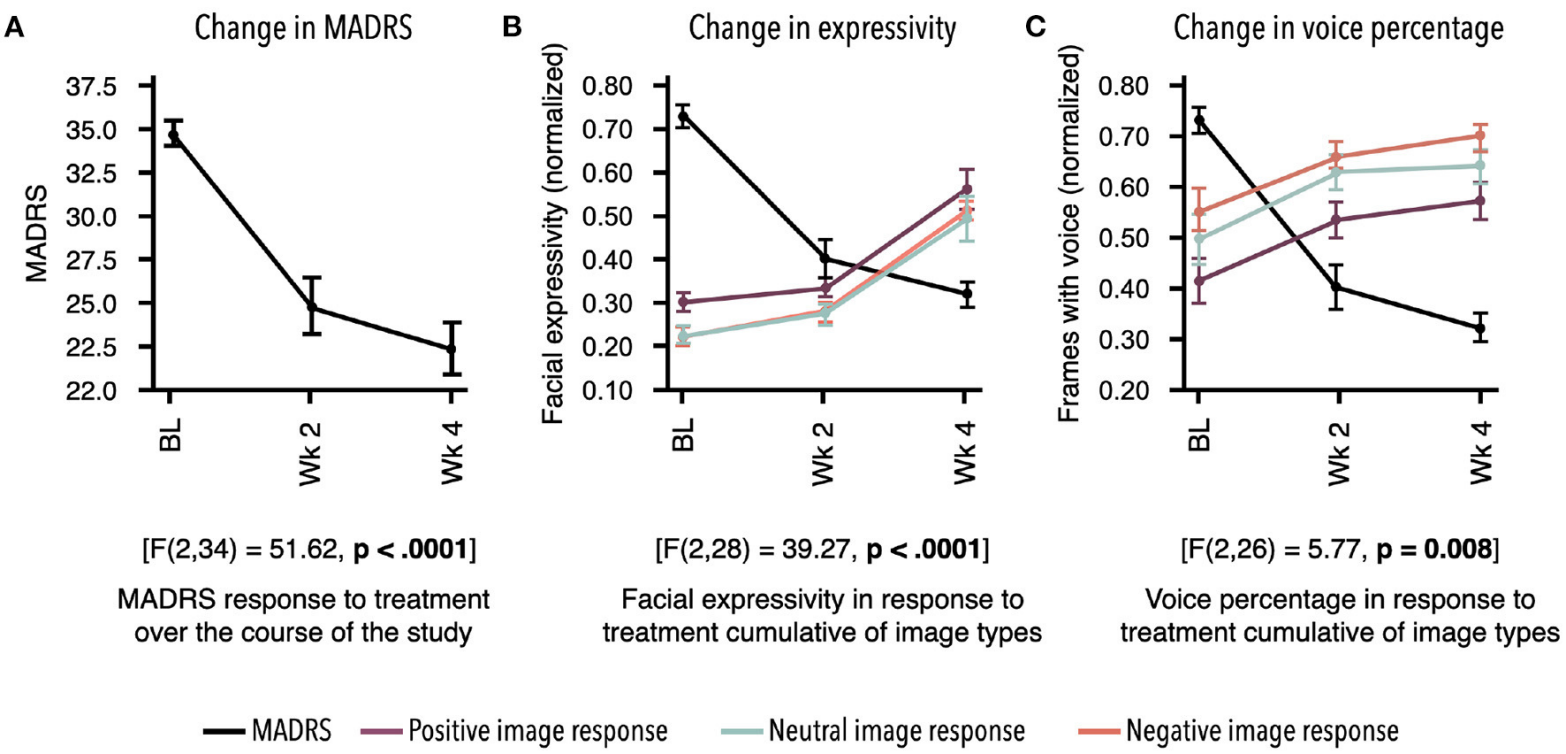
Response to treatment as measured by two independent assessments. Mean for each time point and standard error bars are shown. Results demonstrate treatment's significant effect on digital markers, which are highly concordant with change in depression symptom severity. **(A)** Montgomery–Åsberg Depression Rating Scale (MADRS) scores acquired at baseline (BL), week 2, and week 4 showed significant decrease in response to antidepressant therapy (ADT) [*F*_(2, 34)_ = 51.62, *p* < 0.0001]. **(B)** Overall facial expressivity measured in response to positive [*F*_(2, 28)_ = 40.66; *p* < 0.0001], neutral [*F*_(2, 28)_ = 32.6; *p* < 0.0001], and negative [*F*_(2, 28)_ = 36.95; *p* < 0.0001] images demonstrated a significant increase in response to ADT as MADRS scores decreased. **(C)** Percentage of frames with voice measured in response to positive [*F*_(2, 26)_ = 3.59; *p* = 0.04], neutral [*F*_(2, 26)_ = 5.59; *p* 0.009], and negative [*F*_(2, 28)_ = 4.65; *p* = 0.02] images also demonstrated a significant increase in response to ADT as MADRS scores decreased. All values in **(B,C)** were normalized between 0 and 1.

Participants demonstrated a main effect for change in MADRS scores from baseline to week 4 [*F*_(2, 34)_ = 51.62, *p* < 0.0001]. Descriptive statistics demonstrate clinically relevant change with patients moving from the clinical to non-clinical range (Supplementary Table 1; Figure 2).

Participants demonstrated change in MDD severity as measured by digital markers. To align time points between digital markers and the MADRS scores, measurements from days 7 to 21 were averaged as the week 2 time point, and measurements from days 22to 35 were averaged as the week 4 time point. Due to missed remote assessments, a subset of the total sample of 18 had complete data across time points, with *n* = 12 for facial markers and *n* = 11 for voice markers. All statistical results for digital markers are presented in Table 1. Examples of marker profiles across treatment are presented in Figure 2 alongside the participants' MADRS profile across treatment. All scores, including MADRS, were normalized to a range of 0–1 to allow visual comparison of the magnitude of change on digital markers in comparison with change in MADRS clinical scores (Figure 2).

### Facial Markers

All facial activity measures across all emotions (fear intensity, anger intensity, surprise intensity, sadness intensity, disgust intensity, and overall expressivity) along with the overall expressivity score demonstrated significant positive change from baseline to week 4 in response to all image prompts (positive, neutral, and negative; see Table 1). This result indicates that ADT produces a main effect on facial activity overall, which is not bound to one particular facial musculature group or type of external stimulus (Figure 2).

Across conditions, the frequency of expressions of anger (anger count) decreases. The frequency of expressions of fear also decreases, but only in response to neutral and negative stimuli (fear count). Additionally, the frequency of expressions of happiness (happy count) decreases in response to negative stimuli only. Together, results indicate a general decrease in expressions of anger and context-specific decreases in fear and happiness expressions.

### Voice Markers

The single variable representing the ratio of speech to silence across sentences uttered (voice percentage) additionally demonstrated significant positive change in response to ADT across all conditions, indicating an increase in speech relative to silence. This result is consistent with increased motor/muscle activity observed in facial activity (Figure 2).

### Movement Markers

Additionally, movement parameters demonstrated consistent effects across conditions. The rate of head movement (head movement mean) and the degree of variability in the rate of head movement (head movement standard deviation) both demonstrated significant increases in response to ADT. Head pose change mean also demonstrated significant increase during neutral and positive stimuli (see Table 1).

## Discussion

Results demonstrate a consistent effect of monoamine ADTs (SSRIs/SNRIs) on digital markers of motor functioning, which are highly concordant with change in MDD symptom severity. Specifically, facial and vocal activities demonstrated robust increases across 4 weeks following the initiation of treatment, which mirrored decreases in symptom severity as assessed by the clinician administered MADRS. The current findings suggest that SSRI/SNRI treatment, which produces graded increases in serotonin, reduces depression severity in part by rescuing motor functioning (e.g., increased facial expressivity and increased speech production).

Additionally, a decrease was observed across conditions in the expression of anger. Patients with depression have long demonstrated increased rates of anger than healthy counterparts (44, 45). Furthermore, polymorphisms of the serotonin 1B receptor that are associated with increased depression and suicide risk are also associated with increased anger and fear (46, 47). These results further indicate that the observed change in digital markers in response to serotonin reuptake inhibitors reflects a more specific phenotypic change in measurement of serotonergic profile in the central nervous system.

Serotonin levels in the central nervous system are known to have both direct and indirect effects (*via* dopamine) on motor activity (48, 49). Both suicide (as measured in postmortem brain tissue) and suicidal attempts, a key symptom class of MDD, are associated with depleted serotonin (50). As such, digital measurements that reflect motor behavior may represent a sensitive measure of serotonergic tone and potentially other neurotransmitter activities that affect motor functioning and ultimately the overall clinical presentation.

The current work presents a number of limitations that should be overcome through research that confirms and extends the findings reported. First, while treatment success was confirmed with clinical measures of MDD, dosage and treatment type were not controlled in a manner to make direct inferences about dose–response relationships. Future studies with larger sample sizes that consider different treatment types will have to be conducted to make comparisons on how they might affect digital measurements in varying ways. In addition, the current study was not adequately powered to assess the intra-subject variability in treatment response. Future research should provide more extensive experimental control of medication and dosage to assess the relationship between magnitude of clinical response and digital markers of motor activity.

Second, while facial movement results were robust, we do not know if findings related to specific emotions would rise to significance given a larger sample size or more sampling occasions of the stimuli. One of the goals of the data collection was to implement a very simple remote assessment of objective visual and auditory markers to facilitate ease of frequent assessment. However, the minimum sample to accurately measure each marker needs to be assessed through the use of larger samples. For example, we observed decreases in happiness in response to negative images. This result is difficult to directly interpret. However, given a larger sample, we may be powered to identify increases in happiness in response to positive images, consistent with observations that depressed patients display context-inappropriate affect (51, 52). It is also possible that priming to the stimuli, i.e., the images shown during the remote assessments, was a factor in the behavior recorded and subsequently the data analyzed; both priming to the stimuli and habituation to the assessment need to be evaluated in future work.

Third, subtypes of depression and the range of depression severity observed at the start of treatment were not evaluated as variables in the analysis presented in this study due to the small sample size used. However, the findings observed in this study will greatly inform future work to determine sample sizes needed to measure how digital measurements of facial, vocal, and movement behaviors may differ across subpopulations of MDD as well as different treatment types.

Ultimately, the current work holds promise as an example of the potential to observe treatment effects that reflect underlying neurobiological target engagement by shifting the focus to monotonic neurobiologically based domains rather than heterogeneous diagnoses (6). Further work should determine if these same markers are relevant in other disorders and treatments that are mechanistically affected by serotonergic tone, as well as their relevance to other disorders with motor and movement profiles including Parkinson's disease and schizophrenia (53). Second, the current work demonstrates the success of non-invasive objective digital assessment as a tool to assess treatment effects in MDD, which was the core focus of the study. Importantly, no markers were scientifically novel; rather, they were based on validated methods that are open and public and have been previously reported in scientific literature.

The current work demonstrates, in the context of MDD, that these data sources can be captured remotely through ubiquitously available digital tools to provide measurements that are at least as robust as traditional rating scales. It will be important to determine if such models reliably track with other disease states and treatment responses, as such models and applications have significant potential to increase the rate and accuracy of treatment decision making.

Together, the current study demonstrates that scalability, through digital measurement, of monotonic characteristics that reflects the underlying central nervous system activity. This observation holds promise that frequent remote digital assessment can be used to monitor, titrate, and even personalize treatment for MDD and other psychiatric or neurological conditions by grounding the measurements in narrow phenotypes that match the underlying mechanistic target of the treatment.

## Data Availability Statement

The datasets presented in this study can be found in online repositories. The names of the repository/repositories and accession number(s) can be found at: https://github.com/AiCure/ms_dbm_adamsclinicalstudy.

## Ethics Statement

The studies involving human participants were reviewed and approved by Adams Clinical Institutional Review Board. The patients/participants provided their written informed consent to participate in this study.

## Author Contributions

AA, VY, VK, and IG-L contributed toward the conception of study, development of technology used, data analysis conducted, reporting of results, and writing the manuscript. AA, SM, ME, and CS carried out all participant recruitment, technology onboarding, clinical data collection, and manuscript revisions. All authors contributed to the article and approved the submitted version.

## Conflict of Interest

At the time of the study, AAb, VY, VK and IG-L were employees of AiCure and held stock options in AiCure. CS, AAg, SM, and ME were employees of Adams Clinical, and CS was also an employee of Karuna Therapeutics. The authors declare that the study was jointly funded by AiCure, LLC and Adams Clinical, both of which may benefit from research reported in the manuscript and were involved in study design and execution. Adams Clinical conducted patient recruitment, enrollment, and clinical assessments. AiCure developed methods for digital phenotyping and provided software tools for remote collection of video data used. Both AiCure and Adams Clinical were involved in subsequent data analysis, interpretation, and presentation of findings.

## Supplementary Material

Supplementary Table 1 Means and standard errors across time points of each digital marker in response to neutral, positive and negative stimuli.

Supplementary Table 2 Pairwise comparisons between time points for each digital marker in response to neutral, positive and negative stimuli.

Supplementary Figure 1 Clockwise from top-left, weekly change in digital measurement of overall expressivity, voice percentage, head pose change, and head movement. Each of the variables have been split up by the kind of image that the participants were speaking to: negatively, neutrally, or positively valenced images. In the comparison presented in the main text, values for week 2 and 3 and values for weeks 4 and 5 were averaged into single time points to align the digital measurement time points with the MADRS time points for side-by-side comparison. It also increased the sample size, as not all participants provided consistent weekly data and aggregation across weeks increased the n that could be included in the repeated measures ANOVA. These figures demonstrate the weekly change, further emphasizing the point made in the main text that digital measurements can be conducted with greater frequency than traditional assessments such as the MADRS. However, in these figures, the same patients do not represent each time point. This explains the dip in values is observed at time point 5, which is biased toward the subset of patients that had week 5 data rather than being indicative of a consistent trend.
