# Supplementary material for: Remote Digital Measurement of Facial and Vocal Markers of Major Depressive Disorder Severity and Treatment Response: A Pilot Study
Source: Front Digit Health. 2021 Mar 31;3:610006. doi: 10.3389/fdgth.2021.610006 (PMC8521884; doi:10.3389/fdgth.2021.610006)
Supplement: Supplementary Table 1 — Means and standard errors across time points of each digital marker in response to neutral, positive and negative stimuli. [file Table_1.DOCX]

|  | **Neutral Stimuli** | | | | | | **Positive Stimuli** | | | | | | **Negative Stimuli** | | | | | |
| --- | --- | --- | --- | --- | --- | --- | --- | --- | --- | --- | --- | --- | --- | --- | --- | --- | --- | --- |
|  | *𝜇*  W1 | *S.E.* W1 | *𝜇* W2 | *S.E.* W2 | *𝜇*  W3 | *S.E.* W3 | *𝜇*  W1 | *S.E.* W1 | *𝜇*  W2 | *S.E.* W2 | *𝜇*  W3 | *S.E.* W3 | *𝜇* W1 | *S.E.* W1 | *𝜇* W2 | *S.E.* W2 | *𝜇* W3 | *S.E.* W3 |
| **voiceFrameScore** | 0.49 | 0.04 | 0.60 | 0.03 | 0.64 | 0.03 | 0.41 | 0.03 | 0.52 | 0.04 | 0.57 | 0.04 | 0.54 | 0.03 | 0.64 | 0.03 | 0.70 | 0.02 |
| **angerInt** | 0.28 | 0.01 | 0.30 | 0.02 | 0.46 | 0.04 | 0.30 | 0.02 | 0.33 | 0.02 | 0.47 | 0.03 | 0.34 | 0.01 | 0.37 | 0.02 | 0.54 | 0.04 |
| **angerCount** | 0.08 | 0.02 | 0.15 | 0.05 | 0.03 | 0.01 | 0.09 | 0.04 | 0.13 | 0.04 | 0.05 | 0.02 | 0.05 | 0.02 | 0.13 | 0.05 | 0.04 | 0.02 |
| **disgustInt** | 0.28 | 0.02 | 0.26 | 0.02 | 0.40 | 0.05 | 0.29 | 0.02 | 0.25 | 0.02 | 0.40 | 0.05 | 0.27 | 0.02 | 0.26 | 0.02 | 0.39 | 0.05 |
| **disgustCount** | 0.28 | 0.04 | 0.26 | 0.03 | 0.28 | 0.06 | 0.24 | 0.04 | 0.16 | 0.03 | 0.24 | 0.06 | 0.20 | 0.04 | 0.21 | 0.03 | 0.18 | 0.05 |
| **fearInt** | 0.21 | 0.02 | 0.26 | 0.02 | 0.55 | 0.05 | 0.24 | 0.02 | 0.27 | 0.02 | 0.50 | 0.04 | 0.17 | 0.01 | 0.21 | 0.02 | 0.48 | 0.05 |
| **fearCount** | 0.10 | 0.04 | 0.05 | 0.01 | 0.03 | 0.03 | 0.04 | 0.03 | 0.01 | 0.003 | 0.02 | 0.01 | 0.10 | 0.04 | 0.04 | 0.02 | 0.02 | 0.01 |
| **happyInt** | 0.12 | 0.02 | 0.13 | 0.03 | 0.26 | 0.06 | 0.15 | 0.03 | 0.12 | 0.03 | 0.27 | 0.07 | 0.13 | 0.03 | 0.12 | 0.03 | 0.25 | 0.07 |
| **happyCount** | 0.07 | 0.02 | 0.10 | 0.04 | 0.09 | 0.03 | 0.06 | 0.02 | 0.10 | 0.03 | 0.09 | 0.04 | 0.10 | 0.03 | 0.11 | 0.04 | 0.10 | 0.05 |
| **sadInt** | 0.20 | 0.02 | 0.23 | 0.02 | 0.40 | 0.05 | 0.30 | 0.02 | 0.32 | 0.02 | 0.47 | 0.04 | 0.21 | 0.02 | 0.24 | 0.02 | 0.38 | 0.05 |
| **sadCount** | 0.12 | 0.03 | 0.08 | 0.01 | 0.12 | 0.06 | 0.07 | 0.02 | 0.06 | 0.02 | 0.08 | 0.03 | 0.19 | 0.04 | 0.13 | 0.03 | 0.16 | 0.05 |
| **supriseInt** | 0.41 | 0.03 | 0.42 | 0.05 | 0.69 | 0.04 | 0.42 | 0.03 | 0.42 | 0.04 | 0.65 | 0.04 | 0.39 | 0.03 | 0.42 | 0.05 | 0.66 | 0.04 |
| **supriseCount** | 0.30 | 0.04 | 0.24 | 0.03 | 0.29 | 0.06 | 0.26 | 0.05 | 0.19 | 0.03 | 0.23 | 0.04 | 0.27 | 0.04 | 0.21 | 0.03 | 0.19 | 0.03 |
| **headVelocity** | 0.22 | 0.03 | 0.21 | 0.03 | 0.26 | 0.04 | 0.21 | 0.03 | 0.23 | 0.04 | 0.23 | 0.04 | 0.16 | 0.03 | 0.18 | 0.03 | 0.21 | 0.04 |
| **headVelocitySD** | 0.26 | 0.04 | 0.20 | 0.03 | 0.29 | 0.06 | 0.26 | 0.04 | 0.23 | 0.03 | 0.27 | 0.05 | 0.25 | 0.04 | 0.21 | 0.04 | 0.27 | 0.07 |
| **headPitch** | 0.51 | 0.03 | 0.56 | 0.03 | 0.60 | 0.04 | 0.49 | 0.02 | 0.53 | 0.03 | 0.53 | 0.04 | 0.51 | 0.02 | 0.54 | 0.04 | 0.56 | 0.03 |
| **CompositeInt** | 0.22 | 0.02 | 0.26 | 0.02 | 0.49 | 0.05 | 0.30 | 0.02 | 0.31 | 0.02 | 0.56 | 0.05 | 0.23 | 0.02 | 0.26 | 0.03 | 0.51 | 0.05 |
| *Note*: *𝜇* = mean score; W= week of trial; *S.E.*= standard error of the estimate | | | | | | | | | | | | | | | | | | |
