# Supplementary material for: Remote Digital Measurement of Facial and Vocal Markers of Major Depressive Disorder Severity and Treatment Response: A Pilot Study
Source: Front Digit Health. 2021 Mar 31;3:610006. doi: 10.3389/fdgth.2021.610006 (PMC8521884; doi:10.3389/fdgth.2021.610006)
Supplement: Supplementary Table 2 — Pairwise comparisons between time points for each digital marker in response to neutral, positive and negative stimuli. [file Table_2.docx]

|  | Neutral stimuli | | | | Positive stimuli | | | | Negative Stimuli | | | |
| --- | --- | --- | --- | --- | --- | --- | --- | --- | --- | --- | --- | --- |
|  |  |  | Tukey | |  |  | Tukey | |  |  |  | |
| Variable | margin | SE | T | p | margin | SE | T | p | margin | SE | T | p |
| Voice Percentage  - Week 1 and Week 3  - Week 1 and Week 5  - Week 3 and Week 5 | -0.120  -0.123  -0.003 | 0.068  0.071  0.072 | -1.763  -1.730  -0.044 | 0.185  0.197  0.900 | -0.137  -0.139  -0.001 | 0.071  0.074  0.075 | -1.945  -1.879  -0.019 | 0.129  0.148  0.900 | -0.106  -0.131  -0.024 | 0.059  0.062  0.063 | -1.793  -2.095  -0.375 | 0.175  0.094  0.900 |
| Anger intensity  - Week 1 and Week 3  - Week 1 and Week 5  - Week 3 and Week 5 | -0.052  -0.219  -0.167 | 0.043  0.045  0.046 | -1.196  -4.852  -3.647 | 0.458  0.001  0.001 | -0.072  -0.217  -0.145 | 0.044  0.046  0.047 | -1.625  -4.714  -3.106 | 0.237  0.001  0.006 | -0.052  -0.240  -0.188 | 0.046  0.047  0.048 | -1.132  -5.055  -3.907 | 0.495  0.001  0.001 |
| Anger count  - Week 1 and Week 3  - Week 1 and Week 5  - Week 3 and Week 5 | -0.049  0.028  0.077 | 0.046  0.048  0.049 | -1.051  0.581  1.565 | 0.542  0.813  0.263 | -0.050  -0.004  0.046 | 0.050  0.052  0.053 | -1.007  -0.086  0.866 | 0.568  0.900  0.649 | -0.078  -0.034  0.045 | 0.049  0.051  0.052 | -1.588  -0.656  0.854 | 0.253  0.770  0.656 |
| Disgust intensity  - Week 1 and Week 3  - Week 1 and Week 5  - Week 3 and Week 5 | 0.033  -0.117  -0.150 | 0.054  0.057  0.058 | 0.611  -2.056  -2.601 | 0.796  0.102  0.027 | 0.045  -0.115  -0.160 | 0.055  0.057  0.058 | 0.822  -2.014  -2.760 | 0.674  0.111  0.017 | -0.032  -0.141  -0.109 | 0.054  0.056  0.057 | -0.595  -2.518  -1.917 | 0.805  0.033  0.137 |
| Disgust count  - Week 1 and Week 3  - Week 1 and Week 5  - Week 3 and Week 5 | -0.005  -0.023  -0.018 | 0.079  0.082  0.083 | -0.062  -0.276  -0.213 | 0.900  0.900  0.900 | 0.110  0.033  -0.077 | 0.083  0.087  0.088 | 1.313  0.375  -0.871 | 0.390  0.900  0.646 | -0.056  -0.031  0.025 | 0.074  0.077  0.079 | -0.759  -0.404  0.320 | 0.711  0.900  0.900 |
| Fear intensity  - Week 1 and Week 3  - Week 1 and Week 5  - Week 3 and Week 5 | -0.076  -0.397  -0.321 | 0.051  0.053  0.054 | -1.491  -7.477  -5.953 | 0.298  0.001  0.001 | -0.070  -0.323  -0.254 | 0.049  0.051  0.052 | -1.411  -6.281  -4.851 | 0.337  0.001  0.001 | -0.069  -0.365  -0.296 | 0.048  0.050  0.051 | -1.438  -7.266  -5.795 | 0.323  0.001  0.001 |
| Fear count  - Week 1 and Week 3  - Week 1 and Week 5  - Week 3 and Week 5 | 0.100  0.134  0.034 | 0.062  0.064  0.065 | 1.621  2.086  0.523 | 0.239  0.095  0.847 | 0.015  0.002  -0.014 | 0.016  0.016  0.017 | 0.972  0.094  -0.825 | 0.588  0.900  0.673 | 0.019  0.063  0.045 | 0.056  0.058  0.059 | 0.339  1.096  0.759 | 0.900  0.516  0.711 |
| Happiness intensity  - Week 1 and Week 3  - Week 1 and Week 5  - Week 3 and Week 5 | -0.002  -0.116  -0.114 | 0.057  0.059  0.060 | -0.040  -1.966  -1.898 | 0.900  0.123  0.142 | 0.017  -0.118  -0.135 | 0.068  0.070  0.072 | 0.253  -1.674  -1.887 | 0.900  0.218  0.145 | -0.005  -0.112  -0.107 | 0.058  0.060  0.061 | -0.080  -1.868  -1.763 | 0.900  0.151  0.185 |
| Happiness count  - Week 1 and Week 3  - Week 1 and Week 5  - Week 3 and Week 5 | -0.004  -0.004  0.000 | 0.036  0.037  0.038 | -0.112  -0.097  0.010 | 0.9  0.9  0.9 | -0.019  0.002  0.022 | 0.043  0.045  0.045 | -0.449  0.056  0.479 | 0.889  0.900  0.872 | 0.011  0.042  0.031 | 0.054  0.057  0.058 | 0.202  0.745  0.543 | 0.900  0.719  0.835 |

|  | Neutral stimuli | | | | Positive stimuli | | | | Negative Stimuli | | | |
| --- | --- | --- | --- | --- | --- | --- | --- | --- | --- | --- | --- | --- |
|  |  |  | Tukey | |  |  | Tukey | |  |  | Tukey | |
| Variable | margin | SE | T | p | margin | SE | T | p | margin | SE | T | p |
| Sadness intensity  - Week 1 and Week 3  - Week 1 and Week 5  - Week 3 and Week 5 | -0.020  -0.191  -0.171 | 0.049  0.051  0.052 | -0.414  -3.749  -3.301 | 0.900  0.001  0.003 | 0.012  -0.137  -0.148 | 0.043  0.045  0.046 | 0.270  -3.040  -3.247 | 0.900  0.007  0.004 | -0.065  -0.194  -0.128 | 0.050  0.052  0.053 | -1.309  -3.717  -2.423 | 0.392  0.001  0.043 |
| Sadness count  - Week 1 and Week 3  - Week 1 and Week 5  - Week 3 and Week 5 | 0.011  -0.052  -0.063 | 0.066  0.069  0.070 | 0.166  -0.762  -0.907 | 0.900  0.709  0.625 | -0.046  -0.078  -0.033 | 0.041  0.043  0.043 | -1.115  -1.840  -0.758 | 0.505  0.160  0.711 | -0.004  -0.089  -0.085 | 0.078  0.081  0.082 | -0.050  -1.100  -1.036 | 0.900  0.514  0.551 |
| Surprise intensity  - Week 1 and Week 3  - Week 1 and Week 5  - Week 3 and Week 5 | -0.030  -0.312  -0.282 | 0.077  0.080  0.081 | -0.394  -3.898  -3.465 | 0.900  0.001  0.002 | -0.061  -0.288  -0.227 | 0.069  0.072  0.073 | -0.891  -4.007  -3.104 | 0.635  0.001  0.006 | -0.054  -0.292  -0.238 | 0.078  0.082  0.083 | -0.691  -3.584  -2.876 | 0.750  0.001  0.012 |
| Surprise count  - Week 1 and Week 3  - Week 1 and Week 5  - Week 3 and Week 5 | -0.001  -0.027  -0.025 | 0.073  0.076  0.077 | -0.018  -0.350  -0.327 | 0.9  0.9  0.9 | 0.004  -0.029  -0.033 | 0.071  0.074  0.075 | 0.056  -0.398  -0.445 | 0.900  0.900  0.892 | -0.023  0.005  0.028 | 0.064  0.067  0.068 | -0.361  0.075  0.415 | 0.900  0.900  0.900 |
| Overall expressivity  - Week 1 and Week 3  - Week 1 and Week 5  - Week 3 and Week 5 | -0.037  -0.284  -0.246 | 0.052  0.054  0.055 | -0.722  -5.259  -4.495 | 0.732  0.001  0.001 | -0.032  -0.288  -0.256 | 0.053  0.055  0.056 | -0.609  -5.213  -4.557 | 0.797  0.001  0.001 | -0.059  -0.305  -0.246 | 0.054  0.056  0.057 | -1.096  -5.454  -4.334 | 0.516  0.001  0.001 |
| Head velocity  - Week 1 and Week 3  - Week 1 and Week 5  - Week 3 and Week 5 | 0.008  -0.099  -0.107 | 0.049  0.051  0.052 | 0.164  -1.950  -2.074 | 0.900  0.128  0.098 | 0.016  -0.053  -0.070 | 0.049  0.051  0.052 | 0.333  -1.039  -1.337 | 0.900  0.549  0.377 | -0.026  -0.072  -0.046 | 0.053  0.055  0.056 | -0.481  -1.298  -0.824 | 0.871  0.398  0.673 |
| Standard deviation of head velocity  - Week 1 and Week 3  - Week 1 and Week 5  - Week 3 and Week 5 | 0.021  -0.110  -0.131 | 0.073  0.076  0.077 | 0.287  -1.449  -1.697 | 0.900  0.318  0.209 | 0.030  -0.047  -0.078 | 0.071  0.074  0.075 | 0.429  -0.638  -1.033 | 0.900  0.780  0.552 | -0.028  -0.120  -0.092 | 0.094  0.098  0.099 | -0.294  -1.228  -0.930 | 0.900  0.439  0.612 |
| Head pose  - Week 1 and Week 3  - Week 1 and Week 5  - Week 3 and Week 5 | -0.025  -0.142  -0.116 | 0.070  0.073  0.074 | -0.363  -1.949  -1.576 | 0.900  0.128  0.258 | 0.020  -0.012  -0.031 | 0.020  0.021  0.021 | 0.976  -0.546  -1.459 | 0.586  0.833  0.313 | -0.015  -0.094  -0.079 | 0.082  0.085  0.087 | -0.183  -1.098  -0.908 | 0.900  0.515  0.625 |
