# Supplementary figures and images for: Remote Digital Measurement of Facial and Vocal Markers of Major Depressive Disorder Severity and Treatment Response: A Pilot Study
Source: Front Digit Health. 2021 Mar 31;3:610006. doi: 10.3389/fdgth.2021.610006 (PMC8521884; doi:10.3389/fdgth.2021.610006)

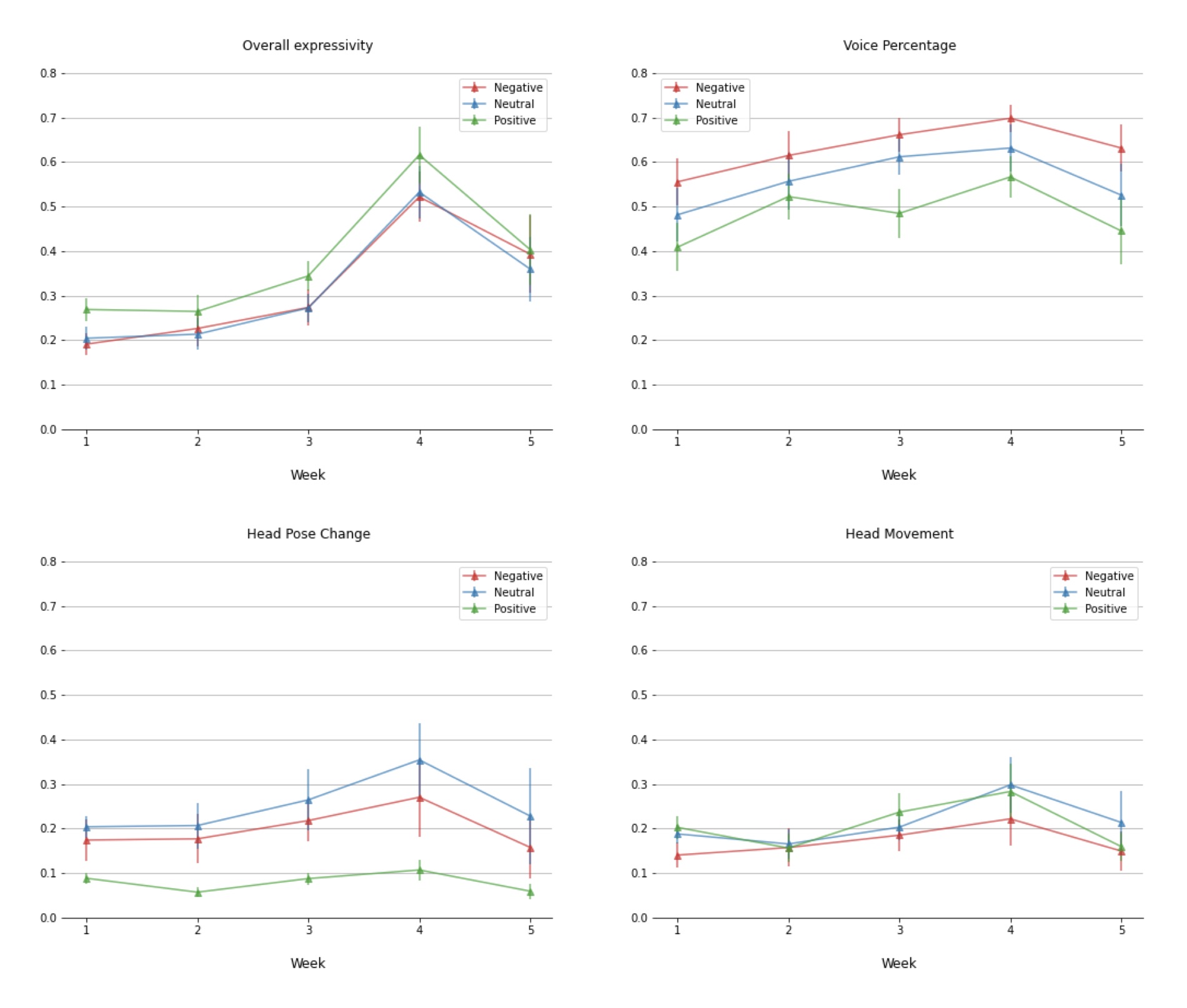

Supplement: Supplementary Figure 1 — Clockwise from top-left, weekly change in digital measurement of overall expressivity, voice percentage, head pose change, and head movement. Each of the variables have been split up by the kind of image that the participants were speaking to: negatively, neutrally, or positively valenced images. In the comparison presented in the main text, values for week 2 and 3 and values for weeks 4 and 5 were averaged into single time points to align the digital measurement time points with the MADRS time points for side-by-side comparison. It also increased the sample size, as not all participants provided consistent weekly data and aggregation across weeks increased the n that could be included in the repeated measures ANOVA. These figures demonstrate the weekly change, further emphasizing the point made in the main text that digital measurements can be conducted with greater frequency than traditional assessments such as the MADRS. However, in these figures, the same patients do not represent each time point. This explains the dip in values is observed at time point 5, which is biased toward the subset of patients that had week 5 data rather than being indicative of a consistent trend. [file Image_1.JPEG]
